# Photobiomodulation of Human Fibroblasts and Keratinocytes with Blue Light: Implications in Wound Healing

**DOI:** 10.3390/biomedicines9010041

**Published:** 2021-01-05

**Authors:** Francesca Rossi, Giada Magni, Francesca Tatini, Martina Banchelli, Federica Cherchi, Michele Rossi, Elisabetta Coppi, Anna Maria Pugliese, Duccio Rossi degl’Innocenti, Domenico Alfieri, Francesco S. Pavone, Roberto Pini, Paolo Matteini

**Affiliations:** 1 Istituto di Fisica Applicata “Nello Carrara”, Consiglio Nazionale delle Ricerche (CNR-IFAC), 50019 Florence, Italy; f.rossi@ifac.cnr.it (F.R.); f.tatini@ifac.cnr.it (F.T.); michele.rossi.od@gmail.com (M.R.); r.pini@ifac.cnr.it (R.P.); p.matteini@ifac.cnr.it (P.M.); 2 Department of Neuroscience, Psychology, Drug Research and Child Health, Section of Pharmacology and Toxicology, University of Florence, 50139 Florence, Italy; federica.cherchi@unifi.it (F.C.); elisabetta.coppi@unifi.it (E.C.); 3 EmoLED s.r.l., Sesto Fiorentino, 50019 Florence, Italy; d.rossi@emoled.com (D.R.d.); rs@emoled.com (D.A.); 4 Department of Physics, University of Florence, 50019 Florence, Italy; francesco.pavone@unifi.it; 5 European Laboratory for Non-Linear Spectroscopy (LENS), 50019 Florence, Italy; 6 Istituto Nazionale di Ottica, Consiglio Nazionale delle Ricerche (CNR-INO), 50125 Florence, Italy

**Keywords:** photobiomodulation, blue light, LED, wound healing

## Abstract

In recent years, photobiomodulation (PBM) has been recognized as a physical therapy in wound management. Despite several published research papers, the mechanism underlying photobiomodulation is still not completely understood. The investigation about application of blue light to improve wound healing is a relatively new research area. Tests in selected patients evidenced a stimulation of the healing process in superficial and chronic wounds treated with a blue LED light emitting at 420 nm; a study in animal model pointed out a faster healing process in superficial wound, with an important role of fibroblasts and myofibroblasts. Here, we present a study aiming at evidencing the effects of blue light on the proliferation and metabolism in fibroblasts from healthy skin and keratinocytes. Different light doses (3.43, 6.87, 13.7, 20.6, 30.9 and 41.2 J/cm${}^{2}$) were used to treat the cells, evidencing inhibitory and stimulatory effects following a biphasic dose behavior. Electrophysiology was used to investigate the effects on membrane currents: healthy fibroblasts and keratinocytes showed no significant differences between treated and not treated cells. Raman spectroscopy revealed the mitochondrial Cytochrome C (Cyt C) oxidase dependence on blue light irradiation: a significant decrease in peak intensity of healthy fibroblast was evidenced, while it is less pronounced in keratinocytes. In conclusion, we observed that the blue LED light can be used to modulate metabolism and proliferation of human fibroblasts, and the effects in wound healing are particularly evident when studying the fibroblasts and keratinocytes co-cultures.

## 1. Introduction

Photobiomodulation (PBM) has been recently recognized as a physical therapy for wound care treatment: a physical therapy is related to the interaction between the wound and a device that delivers energy to the wound; this interaction induces observable and measurable modifications in the wound [1]. The first evidence of photobiomodulation was published in the second half of the 1960s [2]: since then, several applications of infrared (IR) or near infrared (NIR) lasers were studied. With the photonics technology development, new regions of the electromagnetic spectrum were investigated, and new light sources, such as light emitting diodes (LEDs), gained interest for their reduced production costs, compactness and usability in respect to laser sources. In the last 20 years, the technology development brought to the market new LEDs, emitting in a broad wavelength range, including the blue region. These compact and low-cost light sources opened up the possibility to design novel light-based therapies, for the treatment of different pathologies, e.g., several different dermatological conditions, brain injuries and spinal cord damages [3,4,5,6,7]. Despite an increasing number of studies regarding the effect of blue light in tissues and cells, the reported information is often incomplete, and it is hard to have a clear and objective comparison of different approaches [5], therefore the underlying mechanism is not deeply understood. However, the primary and secondary effects of the light in the range 600–1000 nm have been extensively investigated: mitochondria seem to be the principal cellular light photoacceptor and the Cytochrome C (Cyt C) oxidase appears to be the main molecule involved. It is reported that the excitation of the Cyt C activates a cascade of cellular signaling which ends in the modulation of cellular metabolism, proliferation, migration and adenosine triphosphate (ATP) synthesis [5,8,9]. In our previous studies, we reported the observations of an improved healing process when superficial wounds are treated with a blue LED light [10,11,12,13] emitting in the range 410–430 nm. Tests in selected patients [14,15] confirmed the results of the studies in animal models, showing a high closuring rate in hard-to-heal wounds treated with a blue LED light. We thus decided to investigate the interaction of this particular blue irradiation at a cellular level. In the present study, we focused on fibroblast derived from human healthy tissue, as we previously reported in vivo that the activity of fibroblasts is modulated by the blue LED light [16,17]. Moreover, they play an important role in the wound healing process and tissue morphology reconstruction. Here, we also propose the simultaneous use of a well-known cellular model, HaCaT cells, and primary human cell cultures, which gives us the opportunity to compare our result with the literature and at the same time to exploit the complexity of fresh, non-immortalized cell lines. The investigation of cultured keratinocytes cells and the co-culture of fibroblast and keratinocytes gives an overview on a more complex model, better mimicking tissue behavior. The main goal of the present work is to study the effects blue LED (in the range 410–430 nm) in different cells types and with different light doses. The aim is to verify whether this wavelength can induce PBM.

## 2. Materials and Methods

### 2.1. The Device

The blue LED device is based on the use of a commercially available LED, emitting at around 420 nm, 1 W optical emission power and 1.2 W/cm${}^{2}$ power density, as previously described [18]. The light intensity distribution at the tissue surface is homogeneous [12]. During irradiation the fiber tip was put at a constant distance (1 cm) from the cultured cells plate [18]. Different light doses were used to irradiate the cells, by keeping the same power density (680 mW/cm${}^{2}$) and varying irradiation times from 5 to 60 s. The resulting fluences used in this study are 3.43, 6.87, 13.7, 20.6, 30.9 and 41.2 J/cm${}^{2}$. The fluence values were calculated taking into account the dimension of the well and the irradiation spot, at the maximum energy provided by the device. All the irradiation parameters were measured using a photodiode energy sensor (Ophir, Darmstadt, Germany).

### 2.2. Human Keratinocytes Cell Line

Spontaneously transformed non-tumorigenic human keratinocyte cell line (HaCaT) was purchased from Elabscience (Houston, TX, USA). Cells were cultivated in T75 flask (Greiner Bio-One Italia, Milan, Italy) in Dulbecco Modified Eagle Medium (DMEM) high glucose (4.5 g/L) supplemented with 10% Fetal Bovine Serum (FBS) (Pan-React Applichem, Milan, Italy), 1% Glutamine and 1% Penicillin–Streptomycin (pen/strep) (EuroClone, Milan, Italy). Cells were kept at 37 °C and 5% CO${}_{2}$–95% air in humidified atmosphere. The medium was refreshed every 48 h and the cells were split upon reaching 75% confluence.

### 2.3. Human Healthy Skin Samples and Fibroblasts Primary Cultures

The healthy human skin samples were obtained from 7 healthy patients subjected to mole removal. Surgeries were performed at the Azienda Ospedaliera Università degli Studi di Perugia (Italy). The study was approved by the Hospital Ethical Board (16806/19/AV, 07/17/2019). All the experiments were performed in accordance with the Helsinki declaration and in conformity with Good Clinical Practice (GPC). After the biopsy, healthy skin tissues were immediately frozen at −80 °C in DMEM. To prepare primary cultures, the samples were thawed at 37 °C and fragmented into small pieces. Each specimen was collected in a scratched-plated (Greiner Bio-One Italia, Milan, Italy) and kept under laminar flow carefully avoiding the dehydration until the adhesion to the plate occurred [19]. After this procedure, DMEM low glucose (1.5 g/L) medium (Pan-React Applichem, Milan, Italy), supplemented with 10% FBS, 1% Glutamine and 1% pen/strep (EuroClone, Milan, Italy), was added and cells maintained at (37 °C and 5% CO${}_{2}$). Within three weeks from the preparation, fibroblasts migrated out of the tissue. When fibroblasts reached confluence, the cells were detached using Trypsin-EDTA solution (Sigma-Aldrich, Milan, Italy), collected in a centrifuge tube, centrifuged and the pellet was seeded in T25 flask (Greiner Bio-One Italia, Milan, Italy). Fibroblasts were maintained under standard culture conditions (37 °C and 5% CO${}_{2}$) in T75 flask (Greiner Bio-One Italia, Milan, Italy), and the medium was refreshed every 48 h. Cells were split when reaching about 80% of confluence.

### 2.4. Cell Counting Kit-8 and Sulforhodamine B Based Assays

Cell Counting Kit-8 (CCK-8) was used to measure cellular metabolic activity [20,21], while Sulforhodamine B-based assay (SRB) was used to evaluate cell proliferation [22,23,24]. The CCK-8 uses WST-8, which produces a water-soluble formazan dye according to the mitochondrial dehydrogenase activity. Colorless WST-8 is bioreduced by cellular dehydrogenases and becomes WST-8 formazan with an orange color that is soluble in the tissue culture medium. Sulforhodamine binds stoichiometrically to proteins under mildly acidic conditions and then can be extracted under basic conditions; thus, the amount of bound dye can be used as an approximation of cell mass, which can then be extrapolated to measure cell proliferation. Both CCK-8 and SRB were purchased from Sigma-Aldrich (Milan, Italy) and used in according to manufacturer instructions. Fibroblasts and HaCaT cells were counted by using Neubauer chamber (Karl Hecht Assistent GmbH, Sondheim vor der Rhön, Germany) and 5 $\times \;10^{3}$ or 8 $\times \;10^{3}$ cells, respectively, were seeded in appropriate DMEM in distinct 96-well plates (Greiner Bio-One Italia, Milan, Italy). Before the experiments, cells were maintained for 24 h in an incubator upon standard culture conditions (37 °C and 5% CO${}_{2}$). Prior light irradiation, DMEM low or high glucose was replaced with DMEM without FBS and phenol red and the cells were irradiated at different blue LED light doses: 3.43, 6.87, 13.7, 20.6, 30.9 and 41.2 J/cm${}^{2}$. In each experiment, each dose of blue LED light was applied in three separate wells, while three other wells were left not irradiated and used as a control. The absorbance was evaluated using an automatic microplate absorbance reader (LT-4000 Labtech, Heathfield, East Sussex, England), processing the values with specific commercial software (GraphPad Prism, San Diego, CA, USA). Each experiment was performed at least in duplicate. The hypothesis of normality was verified both in HaCaT and in fibroblast cells. Some datasets failed the normality test. Kruskal–Wallis test was used followed by a Dunn’s post-hoc tests (not irradiated cells vs. treated cells).

### 2.5. DAPI and Trypan Blue Analysis

The 4,6-diamidino-2-phenylindole (DAPI) (Sigma-Aldrich, Milan, Italy) was used to label the cellular nuclei to perform an estimation of the nuclear fragmentation induced by the application of 41.2 J/cm${}^{2}$, the maximum dose used in our experiments [25,26]. HaCaT and fibroblast cells were seeded in μ-dish 35 mm with a polymer coverslip bottom glass (Ibidi, GMBH, Martinsried, Germany) to ensure the following confocal analysis. From 24 and 48 h after the irradiation, DMEM was replaced with phosphate buffer saline (PBS) and two washes were performed. After that, cells were fixed for 6 min with a 3.6% solution of paraformaldehyde diluted in PBS. After two washes, one drop of DAPI was added on each sample and coverslips were mounted. The images were acquired with SP8 confocal microscope (Leica Microsystems, Mannheim, Germany), using a 40× water-immersion objective (NA 0.75 Plan). The dye exclusion test was used to label necrotic cells and at the same time, to estimate the number of cells before and after treatment with 41.2 J/cm${}^{2}$ of fluence. In the experiments performed using Trypan Blue solution 0.4% [27,28], cells were seeded in 35 mm petri dishes. Both the presence of necrotic cells and the number of total cells in treated (41.2 J/cm${}^{2}$) and untreated samples were evaluated. After 24 and 48 h, two washes with PBS were performed before the staining. Trypan blue was applied for 6 min, and, after several washes in PBS, ten random images were immediately acquired under an inverted microscope (INV100T) using a 5-megapixel photo-camera (both purchased from Eurotek Orma, Milan, Italy). The collected images were analyzed with open-source software (ImageJ, version 1.49v National Institutes of Health, Bethesda, MD, USA) by two trained operators. The experiment was performed in triplicate. In both cases, the treatments were performed in DMEM high or low glucose, without FBS and red phenol, to rule out both experimental bias and interference during the irradiation. Data are expressed as mean ± SEM (standard error of the mean). Student’s paired two-tailed *t*-tests was performed, as appropriate, in order to determine statistical significance. Data were analyzed using software package GraphPad Prism (GraphPad Software, San Diego, CA, USA).

### 2.6. Electrophysiological Recordings

Whole-cell patch-clamp recordings were performed in −60 mV clamped-cells as described [18,29]. The following solutions were used. Extracellular solution (mM): NaCl 147, KCl 4, MgCl${}_{2}$ 1, CaCl${}_{2}$ 2, HEPES (4-(2-hydroxyethyl)-1-piperazine ethanesulfonic acid) 10, D-glucose 10 (pH 7.4 with NaOH). Standard K${}^{+}$-based pipette solution (mM): K-gluconate 130, NaCl 4.8, KCl 10, MgCl${}_{2}$ 2, CaCl${}_{2}$ 1, Na${}^{2}$-ATP 2, Na${}^{2}$-GTP 0.3, EGTA 3, HEPES 10 (pH 7.4 with KOH). Cells were plated into 13 mm diameter coverslips and allowed to adhere before starting the recordings. Each sample was transferred to a 1 mL recording chamber mounted on the platform of an inverted microscope (Olympus CKX41, Milan, Italy) superfused at a flow rate of 1.5 mL/min by a three-way perfusion valve controller (Harvard Apparatus). Borosilicate glass electrodes (Harvard Apparatus, Holliston, MA, USA) were pulled with a Sutter Instruments puller (model P-87) to a final tip resistance of 3–5 MΩ. Data were acquired with an Axopatch 200B amplifier (Axon Instruments, Union City, CA, USA), low-pass filtered at 10 kHz, stored and analyzed with pClamp 9.2 software (Axon Instruments, Union City, CA, USA). A depolarizing voltage ramp protocol (800 ms depolarization from −80 to +80 mV for HF and from −80 to +120 mV for HaCat) was used to activate overall voltage-dependent currents in these cells. Pilot experiments performed by us revealed that a voltage ramp to +80 mV is able to activate the majority of voltage-dependent conductances in human fibroblasts whereas, in HaCaT cells, a +120 mV is needed to achieve similar levels of current amplitudes. In fact, at +80 mV, the steepest tract of the ramp traces in HaCaT cells is still to be evoked. It was repeated every 15 s to evoke overall voltage-dependent currents before, during and after the application of 20.6 J/cm${}^{2}$ of blue LED light. Total outward currents evoked by the voltage ramp were quantified by measuring current amplitude at +80 mV for HF and at +120 mV for HaCat. Control values were obtained by averaging the last 4 traces (1 min) of baseline and were compared to those measured during the fifth minute after irradiation. In averaged results, current amplitude (in pA) was normalized to cell capacitance (in pF) and expressed as pA/pF. The experiments were performed on 7 fibroblasts and 6 HaCaT cells.

### 2.7. Micro Raman Measurements on Single Cell

Raman experiments were carried out on a micro-Horiba Xplora coupled to a 532 nm wavelength laser for the excitation. The spectrograph used 1200 grooves mm${}^{-1}$ grating with a confocal microscope in backscattering geometry and a 2D-CCD camera. The backscattered light was collected by a 50× microscope objective with 0.80 NA, which generated a ≈ 2 μm large laser beam waist. An integration time of 5 s and a laser power value of ∼1 mW on the sample were employed for Raman measurements on fibroblast and HaCaT cells. Pellets were prepared by two centrifugations in PBS at 1000 rpm for 6 min and removal of the supernatant. Typically, for a single Raman measurement, a volume of 2 μL of the pellet was drop-casted onto a gold mirror support (ME1S-M01; Thorlabs, Inc., Newton, NJ, USA), and Raman spectra were immediately recorded on samples before the blue LED light irradiation (*n* = 3) and after the application of 20.6 J/cm${}^{2}$ (*n* = 3) and 41.2 J/cm${}^{2}$ (*n* = 3) of blue LED irradiation treatment. This procedure enabled to maintain the same experimental condition in control and treated samples. For each sample, at least 20 individual cells were inspected while carefully avoiding cell dehydration. All data were baseline corrected when needed and normalized to the peak of Phenylalanine. As a reference, the Raman spectrum of an aqueous solution of Cyt C at 0.1 mM concentration was measured before and after the irradiation with blue LED light. Power laser intensity and integration time in the Raman experiment were optimized to avoid any possible photoreduction or photooxidation of Cyt C during the laser excitation.

### 2.8. In Vitro Co-Culture Scratch Assay

The scratch test assay or wound healing assay is an in vitro procedure used to evaluate the effects of different stimuli on cell migration. In this study, co-culture of HaCat cells and dermal fibroblasts was used to assess if blue LED light can modulate cell migration through this simple preclinical model of wound healing [30,31]. An equal number of HaCaT and fibroblast cells were cultivated in T75 flask (Greiner Bio-One Italia, Milan, Italy) before the experiments. To perform the scratches, cells were plated in 35 mm dishes and scratch wound was made mechanically with a sterile 200 μL tip when complete confluence was reached. After that, the plate was kept in an incubator with humidified atmosphere (37 ${}^{{}^{\circ}}$C and 5% CO${}_{2}$). At different time points (t0, t24, t48 and t72), 2 images at inverted optical microscope (INV100T, Eurotek Orma, Milan, Italy) using 4X objective were acquired. The scratch closure was analyzed by ImageJ (version 1.49v National Institutes of Health, Bethesda, MD, USA), using the “area method”. The scratch area was measured as the ratio between the scratch area at different time points (t24, t48 and t72) and t0, its initial area, multiplied by 100 [30]. The same experimental protocol was used in healthy fibroblast cultures.

## 3. Results

### 3.1. Blue Led Light Affects Metabolism and Proliferation in Both HaCaT Cells and Human Fibroblasts

**Figure 1:**
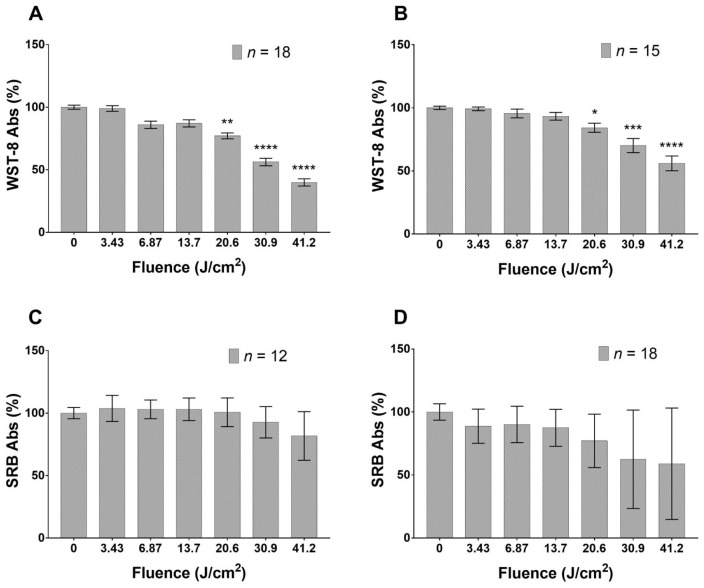
Effects of blue LED light on metabolism and proliferation of HaCaT cells: (**A**,**B**) cell metabolism 24 and 48 h after treatment, respectively; and (**C**,**D**) proliferation 24 and 48 h after treatment, respectively. Data are expressed as mean ± SD. Each measure is repeated in duplicate for each condition. Statistical analysis: * *p* < 0.05; ** *p* < 0.01; *** *p* < 0.001; **** *p* < 0.0001 vs. control (not irradiated cells), Kruskal–Wallis test followed by Dunn’s post-hoc test.

**Figure 2:**
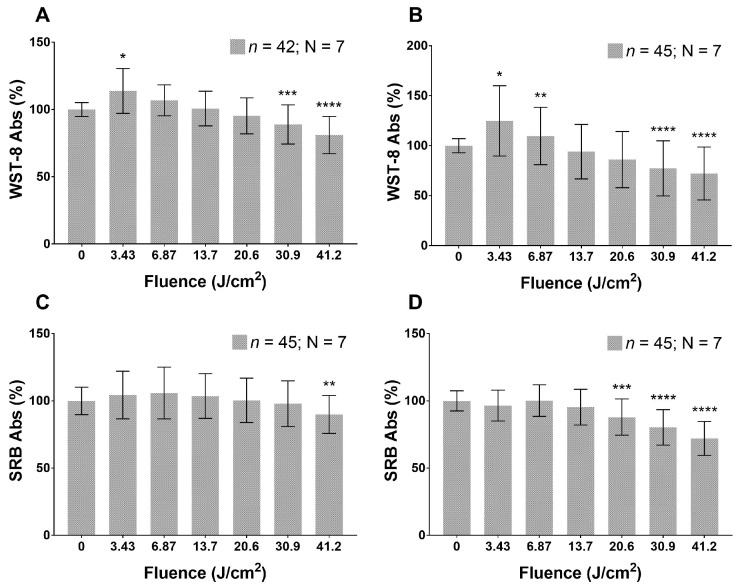
Effects of blue LED light on metabolism and proliferation of fibroblasts cells: (**A**,**B**) metabolism 24 and 48 h after treatment, respectively; and (**C**,**D**) proliferation 24 and 48 h after treatment. Data are expressed as mean ± SD. Each measure is repeated in triplicate; *n*, number of replicates; N, number of human samples. Statistical analysis: * *p* < 0.05; ** *p* < 0.01; *** *p* < 0.001; **** *p* < 0.0001 vs. control (not irradiated cells), Kruskal–Wallis test followed by Dunn’s post-hoc test.

Cultured HaCaT cells reduce their metabolism in a dose-dependent manner 24 h after the application of blue LED light and this trend remains steady at least for the following 24 h. The reduction in cell metabolism reaches significant values when applying fluence doses in the range 20.6–41.2 J/cm${}^{2}$ (Figure 1A,B). As regards proliferation rate (Figure 1C,D), no applied fluence significantly reduces this parameter. The irradiated fibroblasts (Figure 2) show a dual response to blue light. Indeed, the lowest dose (3.43 J/cm${}^{2}$) stimulates an increase in metabolic activity, while fluences of 20.6, 30.9 and 41.2 J/cm${}^{2}$ reduce cell metabolism (Figure 2A) after 24 h from the blue LED light application. Forty-eight hours from the irradiation, a similar effect is also exhibited and becomes more pronounced (Figure 2B). Proliferation shows a reduction only at the highest dose, 24 h from the application, while doses ranging from 20.6 to 41.2 J/cm${}^{2}$ can affect fibroblasts proliferation 48 h after the irradiation (Figure 2C,D).

### 3.2. Blue Led Light Effects on Cell Viability

**Figure 3:**
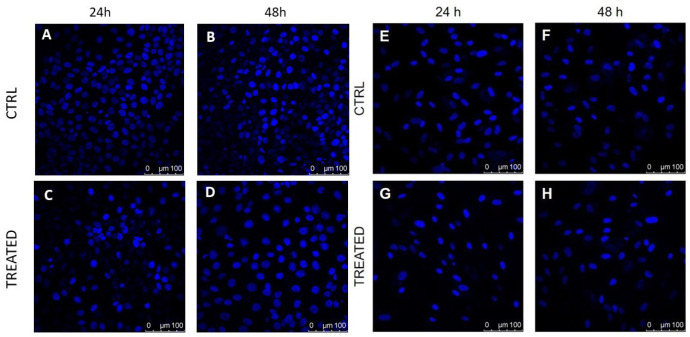
DAPI staining performed on cultured human fibroblasts (right) and HaCaT cells (left) shows differences in treated and control samples: (**A**,**B**) control HaCaT samples analyzed 24 and 48 h after the beginning of the experiment; (**C**,**D**) treated HaCaT cells analyzed at 24 and 48 h after the application of blue light with 41.2 J/cm${}^{2}$ fluence; (**E**,**F**) control fibroblasts samples analyzed 24 and 48 h after the beginning of the experiment; and (**G**,**H**) treated fibroblasts cells analyzed at 24 and 48 h after treatment with a blue light dose of 41.2 J/cm${}^{2}$, respectively. Cells nuclei are in blue.

Trypan blue staining was performed both in fibroblasts and in keratinocytes after the application of 41.2 J/cm${}^{2}$ fluence dose of blue LED light. Table 1 reports values of cell viability before and after irradiation; the hypothesis of normality was verified using the Kolmogorov–Smirnov test. Our results demonstrate that the highest fluence value of blue light significantly reduces the number of total HaCaT cells (observations at 24 and 48 h after the treatment). We observed a significant reduction of cell viability in the fibroblasts cultures 24 h after the treatment, while, at 48 h, no significant differences were found in respect to untreated samples. Qualitative DAPI staining performed in HaCaT cells (Figure 3, left) showed a marked reduction in the number of cells and some morphological differences, which was further investigated in the treated samples versus controls. On the other hand, fibroblasts (Figure 3, right) did not show any significant morphological variation in control and treated samples.

### 3.3. Effects of Blue Led Light on Voltage Dependent Currents in Human Fibroblasts and HaCaT Cells

**Figure 4:**
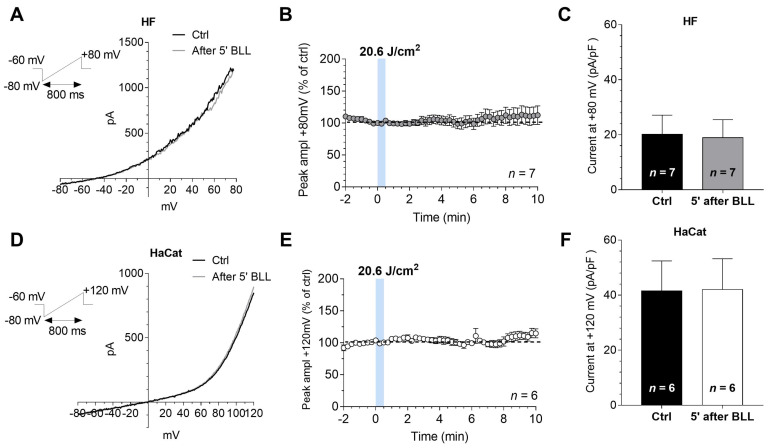
The application of blue LED light does not modify ramp-evoked currents in both cultured fibroblasts and HaCat cells: (**A**) original whole-cell patch clamp current traces evoked by a voltage ramp protocol (from −80 to +80 mV, 800 ms, top left inset) before (Ctrl, black trace) or 5 min after the application of 20.6 J/cm${}^{2}$ blue LED light (BLL, grey trace) in a typical fibroblast (HF); (**B**) averaged time courses (mean ± SEM) of ramp-evoked currents at +80 mV, expressed as percent of ctrl, in HFs (*n* = 7) before, during or after the application of blue LED light; (**C**) pooled data (mean ± SEM) of ramp current amplitude at +80 mV, recorded before or after 5 min of blue LED light (BLL) application in HFs (*n* = 7); (**D**) original whole-cell patch clamp current traces evoked by a voltage ramp protocol (from −80 to +120 mV, 800 ms, top left inset) before (Ctrl, black trace) or 5 min after the application of 20.6 J/cm${}^{2}$ blue LED light (BLL, grey trace) in a typical HaCat cell; (**E**) averaged time courses (mean ± SEM) of ramp-evoked currents at +120 mV, expressed as percent of ctrl, in HaCat cells (*n* = 6) before, during or after the application of 20.6 J/cm${}^{2}$ fluence of blue LED light; and (**F**) pooled data (mean ± SEM) of ramp current amplitude at +120 mV, recorded before or after 5 min of blue LED light (BLL) application in HaCat (*n* = 6).

Recently, we demonstrated that the application of blue LED light, at the dose of 20.6 J/cm${}^{2}$, significantly increased outward currents only in keloid fibroblasts, without effects on fibroblasts isolated from perilesional tissues [32]. These results may support the involvement of voltage activated currents in blue LED light effects. On this basis, by performing patch-clamp recordings, we evaluated the actions of blue LED light on voltage-dependent currents in human fibroblasts isolated from healthy tissues. We decided to verify the effects of light at the same dose used in previous work [32]. Electrophysiological recordings were performed on seven cells showing, on average, a Cm of 54.33 ± 16.30 pF and a Rm of 416.5 ± 142.0 MΩ. In a typical cultured human fibroblast, the application of blue LED light (20.6 J/cm${}^{2}$) did not modify the magnitude of currents evoked by a voltage ramp protocol (from −80 to +80 mV, Figure 4A, top left inset). This protocol allowed simultaneously monitoring the possible modulation of inward (at −80 mV) and outward (at +80 mV) currents by light. No effects of blue LED light were observed on averaged current amplitude evoked by the ramp at +80 mV in seven cells tested (Figure 4B). These effects were similar to those observed in perilesional fibroblasts [32]. Increasing light dose again, no effects were observed (41.2 J/cm${}^{2}$, *n* = 4, data not shown). Pooled data in Figure 4C summarize the panel of responses obtained before and 5 min after irradiation. According to the absence of light effect on ionic currents, no changes in membrane potential was found: on average −62.69 ± 5.98 mV before and −61.3 ± 7.37 mV at 5 min from the end of irradiation (*n* = 7). We further investigated the effects of the same dose of blue LED light (20.6 J/cm${}^{2}$) in HaCaT cells, the immortalized human keratinocytes, extensively used to study the epidermal homeostasis and its pathophysiology. HaCaT cells showed, on average, a Cm of 24.13 ± 4.57 pF and a Rm of 777.4 ± 254.8 MΩ. Similarly, no significant changes in the amplitude of voltage-activated currents, at the more depolarized potential achieved by the voltage ramp (+120 mV) were observed (Figure 4D). In line with the above data, no changes in HaCaT membrane potential were noted. Indeed, immediately before light application Vm was of −29.00 ± 8.24 mV and at 5 min from the end of irradiation, it was −26.92 ± 8.46 mV. Pooled data in Figure 4E summarize the panel of responses obtained before and 5 min after irradiation in 6 HaCat cells.

### 3.4. Raman Microspectroscopy on Irradiated Healthy Fibroblast and HaCaT Cells Revealed Effects of the Blue Light on Cytochrome C Molecule

**Figure 5:**
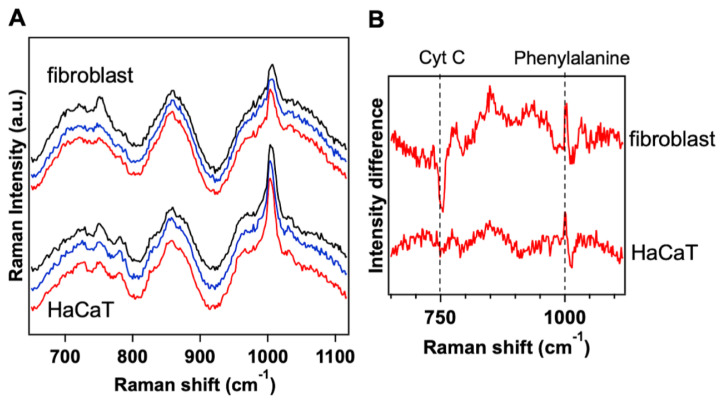
The Raman intensity of Cytochrome C peak at 750 cm${}^{-1}$ undergoes significant variation in fibroblast cells and minor variation in HaCaT cells upon blue LED light irradiation: (**A**) averaged Raman spectra acquired at least on 20 fibroblasts and HaCaT cells before the treatment (black) and after 20.6 J/cm${}^{2}$ (blue) and 41.2 J/cm${}^{2}$ (red) of blue LED light; and (**B**) differential Raman spectrum obtained by subtracting the spectrum acquired before the treatment with blue light from the spectrum acquired after the application of blue light at 41.2 J/cm${}^{2}$, both for cultured fibroblast and HaCaT cells. Phenylalanine mode at 1003 cm${}^{-1}$ was used for normalizing the Raman spectra of the cells.

The Raman spectrum of cultured fibroblasts and HaCaT cells was measured in three different conditions: before the light illumination, after the application of the blue light at 20.6 J/cm${}^{2}$ and after the application of the blue light at 41.2 J/cm${}^{2}$ (Figure 5A). Upon treatment, changes in the Raman spectra of the cells were found and ascribed to molecular changes in Cyt C. These spectral variations were time-dependent, slightly arising soon after irradiation and becoming more visible 20 min after the irradiation. Figure 5A (blue and red curves) shows the effects of blue light on Raman spectrum, which are almost invariant with the applied doses both in fibroblasts and HaCaT cells. To inspect more accurately the spectral variations induced by blue light in the two cell types, the Raman spectrum acquired before the irradiation was subtracted from the spectrum acquired after the irradiation. Figure 5B shows the differential Raman spectrum of fibroblasts and HaCaT cells irradiated with 41.2 J/cm${}^{2}$. The negative intensity of the differential signal at 750 cm${}^{-1}$ for the fibroblasts reveals that the blue light induced a decrease in the Cyt C peak intensity. We recently found that, in fibroblasts from keloid tissue, the blue light produces an intensity increase in the Raman peaks associated to Cyt C [18,32]. Since the intensity of the 750 cm${}^{-1}$ Raman peak of Cyt C is strictly related to the redox state of the molecule, the variation that we ascertained can be ascribed to a significant change in the ratio between the oxidized and reduced form of Cyt C. A change in the redox properties of Cyt C could trigger a cascade of events in the cell, for instance by activating or deactivating redox reactions within the electron transport chain and the respiratory complexes [5]. On the other hand, as clearly shown in Figure 5B, only minor variations in the Raman signals of HaCaT cells were revealed, indicating that this kind of cells are less sensitive to the blue light irradiation.

### 3.5. Blue Led Light Increases Fibroblasts and Keratinocytes Migration in In Vitro Scratch Test

**Figure 6:**
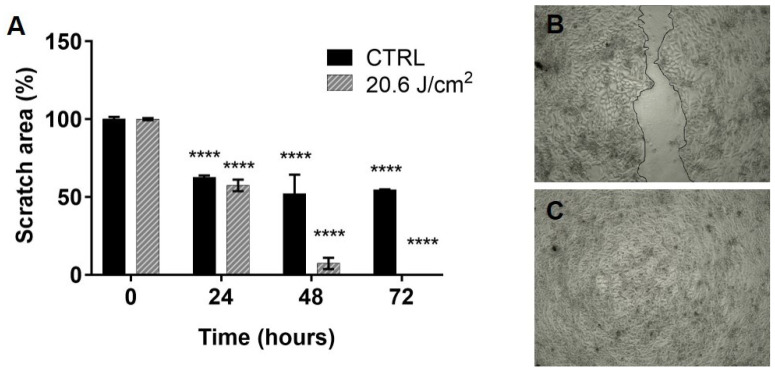
Blue LED light stimulates in vitro fibroblasts and keratinocytes migration: (**A**) column bars represent the percentage of scratch area (i.e., mimicking the wound area) in co-cultures observed 24, 48 and 72 h after treatment, in comparison to 0 h.; and (**B**,**C**) representative images of an untreated (top) and treated (bottom) co-culture of HaCaT and fibroblast, as observed 72 h after treatment (fluence: 20.6 J/cm${}^{2}$). Data are expressed as mean ± SD, *n* = 2. Statistical analysis: **** *p* < 0.0001, t24; t48; t72 vs. t0, one-way ANOVA followed by Dunnett’s multiple comparison test.

Preliminary scratch test performed in co-cultures of HaCaT cells and fibroblasts demonstrated that a fluence of 20.6 J/cm${}^{2}$ can stimulate cellular migration in comparison to untreated sample (Figure 6A). Indeed, the scratch area was significantly reduced by almost 50% in both control and treatment, already 24 h after the beginning of the experiment (0 h). This reduction remained stable in the control sample until 72 h (Figure 6B), while, in the treated sample, the scratch area was further reduced until it closed completely (Figure 6C). When the scratch test was performed in fibroblasts cultured alone, no significant differences between treated and control were found (data not shown).

## 4. Discussion

The main goal of the present study was to investigate the effects of the blue light in the range 410–430 nm at a cellular level and at different doses, in order to further analyze the macroscopic effects observed in our previous studies of wound healing in mice models. The selected cellular target are fibroblasts and keratinocytes, as in our studies in animal models their activity was affected by the blue light treatment. Raman spectroscopy evidenced that the fibroblast Cyt C redox state is significantly affected by blue light irradiation. This is in accordance to the light absorption properties of the Cyt C (it contains the heme group, absorbing in the blue range of the spectrum) and to the hypothesis of PBM mechanism reported in the literature [5,8,9]. PBM is a very complex process that is still under investigation. It is composed of primary and secondary effects: Cyt C oxidase is one of the primary effects, recognized as responsible of PBM in cells. The effect on Cyt C in keratinocytes is less pronounced. Tests performed on cell metabolism and proliferation at different doses evidenced a biphasic dose curve for fibroblasts: lower doses induce an enhancement in fibroblast activity, while higher doses are inhibitory. A similar effect is reported in the literature, and it is observed in cultured cells treated with a different wavelength but in a comparable dose range [5]. Keratinocytes seem to be more sensitive to higher doses, while the effects at low doses are negligible. This different behavior of the two different cell types is particularly clear when we observe the range of viability in cultures treated with higher light doses (41.2 J/cm${}^{2}$): keratinocytes are reduced in number within 24 h after treatment, while there is no difference between treated and control in fibroblast cultures. Finally, a wound healing assay test was designed to further investigate potential harmful effects of blue light in cultured cells: we confirmed results from the literature [33], evidencing that the scratch test in a cultured fibroblast plate is not significantly different in treated and not treated samples. However, in the present study, we evidenced that, when fibroblasts are cultured with HaCaT cells, the healing process is improved and the scratch area is closed within 72 h. Electrophysiology did not evidence a significant role of membrane currents, pointing out a different behavior in respect to fibroblast from keloid tissues [32,34].

## 5. Conclusions

At the beginning of 2010s, we proposed a photocoagulator device, based on the use of a blue LED light emitting in the range 410–420 nm, for the treatment of superficial bleeding wounds. The underlying mechanism of the photocoagulator is a photothermal effect, due to the selective absorption of the blue light by the hemoglobin (and in particular the heme group) in the bleeding wound. Indeed, hemoglobin shows narrow absorption peaks in the blue range, i.e., at 410 and 430 nm for oxygenated and non-oxygenated hemoglobin, respectively. These absorption peaks were used to ensure local temperature increase able to induce hemostasis through a photo-thermo-coagulation process [35]. The use of the proper irradiation settings in terms of dose or fluence, irradiation time, target spot area and emission pattern allows inducing a local temperature rise above the threshold for protein denaturation within the blood, resulting in fast coagulation effect [36]. In the experimental studies in animal models, we used the following parameters: the illuminated area was a circle with 5 mm diameter, a power density of 1.27 W/cm${}^{2}$ and a treatment time of 25 s (resulting in a fluence of 31.7 J/cm${}^{2}$). As a result, a predominant thermal effect inducing coagulation of the bleeding wound was measured. However, the follow up study pointed out an improvement of the healing process, with an apparent modulation of the fibroblast activity and better recovery of the collagen content in the wound area [17]. Further studies in murine model enabled us to optimize treatment conditions to obtain a better healing process, testing lower fluences (21 J/cm${}^{2}$, 680 mW/cm${}^{2}$ and 30 s treatment time) and thus reducing the photothermal effects. The results evidence a modulation of the inflammatory infiltrate and of the cytokines release [16]. The first studies in selected human patients [14,15] pointed out effects in the healing of hard-to-heal wounds that can be ascribed to a photobiomodulation process, with a negligible photothermal effect (irradiation parameters in patients: 120 mW/cm${}^{2}$, 60 s irradiation time and 7.2 J/cm${}^{2}$ fluence). The present work, performed at a cellular level, evidenced that the wound healing promotion effect observed in patients and animal models can be ascribed to a PBM effect in the tissue, even in this particular wavelength range. We observed that the blue light in the range 410–430 nm, delivered to cultured cells in a dose range 3.43–41.2 J/cm${}^{2}$, is able to modulate cells metabolism and proliferation. The response is different depending on cell types and it is dose dependent. However, the biphasic dose response of fibroblast and the induced changes in their redox state confirm the hypothesis that the blue light is able to induce PBM in wounds, as these are the typical PBM effects in cells reported in the literature [5]. Even if blue light does not propagate in the deep tissue, when used in superficial wounds, it transfers the energy to the whole wound area, thus resulting in a predominant photochemical effect possibly stimulating fibroblast activity and promoting the wound healing process.

## Figures and Tables

**Table 1:** Pooled data of cell viability in HaCaT and fibroblast cells at 24 and 48 h after the application of 41.2 J/cm${}^{2}$. Data are expressed as mean and SD (in brackets), two-sample two-tailed *t*-test.

Sample	% of Viable Cells in Ctrl (Not Irradiated Cells)	% of Viable Cells in Irradiated Cells (41.2 J/cm${}^{2}$ Applied)	*p* Value
HaCaT 24 h	224.25 (89.64)	126.42 (100.64)	<0.001
HaCaT 48 h	189.35 (104.80)	61.42 (44.86)	<0.001
Fibroblast 24 h	93.20 (20.05)	44.38 (21.84)	<0.001
Fibroblast 48 h	39.52 (17.08)	38.65 (16.51)	0.778

## Data Availability

The data presented in this study are available on request from the corresponding author. The data are not publicly available due to PI issues and privacy.

## Abbreviations

The following abbreviations are used in this manuscript:

PBM	Photobiomodulation
IR	InfraRed
NIR	Near InfraRed
LED	Light Emitting Diode
Cyt C	Cytochrome C
GPC	Good Clinical Practice
DMEM	Dulbecco Modified Eagle Medium
HaCaT	Spontaneously transformed non-tumorigenic human keratinocyte cell line
FBS	Fetal Serum Bovine
pen/strep	Penicillin/streptomycin
CCK-8	Cell Counting Kit-8
SRB	Sulforhodamine B based assay
DAPI	4,6-diamidino-2-phenylindole
PBS	phosphate buffer saline
